# Distinct Cell Transcriptomic Landscapes Upon Henipavirus Infections

**DOI:** 10.3389/fmicb.2020.00986

**Published:** 2020-05-19

**Authors:** Mingyue Chen, Mary Tachedjian, Glenn A. Marsh, Jie Cui, Lin-Fa Wang

**Affiliations:** ^1^Key Laboratory of Fermentation Engineering, National 111 Center for Cellular Regulation and Molecular Pharmaceutics, Hubei University of Technology, Wuhan, China; ^2^CAS Key Laboratory of Molecular Virology and Immunology, Institut Pasteur of Shanghai, Chinese Academy of Sciences, Shanghai, China; ^3^Center for Biosafety Mega-Science, Wuhan Institute of Virology, Chinese Academy of Sciences, Wuhan, China; ^4^Australian Animal Health Laboratory, CSIRO Health and Biosecurity, Geelong, VIC, Australia; ^5^Programme in Emerging Infectious Diseases, Duke-NUS Medical School, Singapore, Singapore

**Keywords:** bats (Chiroptera), Hendra virus (HeV), Cedar virus, transcriptomatics, cell infection

## Abstract

Hendra virus (HeV) and Cedar virus (CedV) are henipaviruses, which fall into the *Paramyxoviridae* family of single-stranded, negative-sense RNA viruses. HeV is classified as a Biosafety Level-4 (BSL-4) agent, as it is highly pathogenic and is often fatal to humans. To date, no HeV prevention or treatment methods for human are available. In contrast, previous experimental infection studies have suggested that CedV is non-pathogenic. Flying foxes (pteropid bats) in Australia are the natural reservoirs of both viruses, but the cellular responses of bats to these viral infections remain unclear. Here, we infected bat and human cells with these viruses. We then examined the total transcriptomic landscapes of the cells at 6 or 24 h post infection. Unexpectedly, despite the close phylogenetic relationship between HeV and CedV, there was a dramatic difference in cellular gene expression patterns in response to the two different infections. It is likely that minor differences in the phosphoprotein (P) gene coding strategy between the two viruses cause the observed incongruence in host transcriptomic divergence and viral lethality. This study greatly expands our understanding of the pathogenic mechanisms of henipaviruses.

## Introduction

**FIGURE 1:**
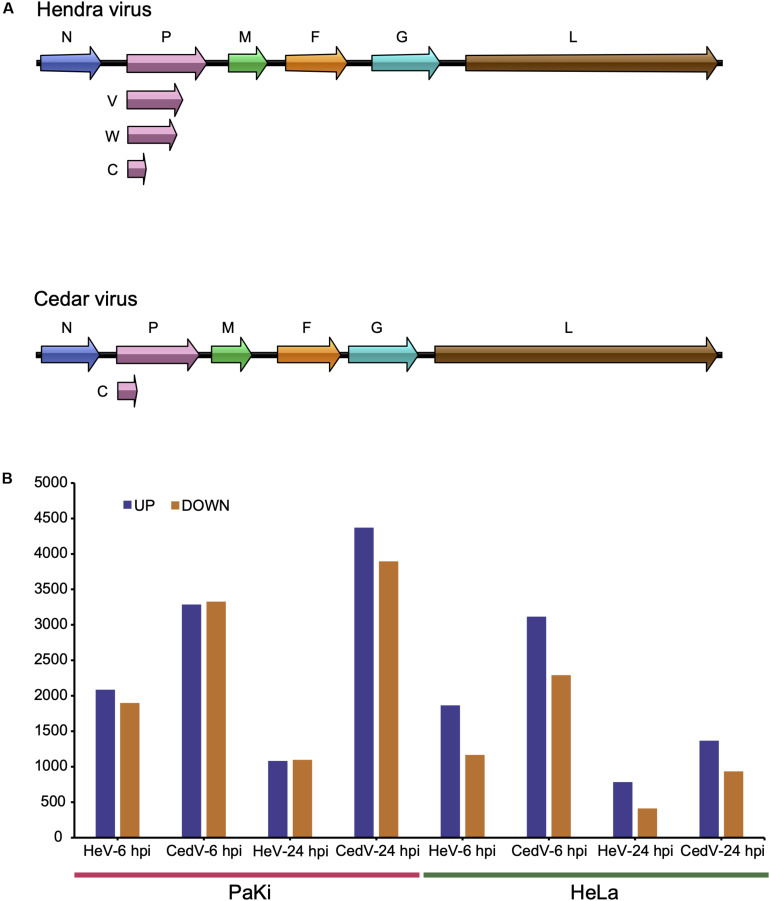
HeV and CedV genome organization and RNA-seq profiles**. (A)** Genome organization of HeV and CedV. **(B)** DEGs in HeV- or CedV-infected HeLa and PaKi cells at 6 and 24 hpi. HeV-6 hpi and CedV-6 hpi represent HeV- or CedV-infected corresponding PaKi or HeLa at 6 hpi. HeV-24 hpi and CedV-24 hpi represent HeV- or CedV-infected corresponding PaKi or HeLa at 24 hpi.

Two viruses in the genus *Henipavirus* (Eaton et al., 2006; Marsh et al., 2012), Hendra (HeV) and Nipah (NiV), are the only known Biosafety Level 4 (BSL-4) pathogens in the family *Paramyxoviridae* (Marsh et al., 2012). Both of these viruses cause 40–100% mortality in humans and other animals (Eaton et al., 2006). The natural reservoir of HeV is the Australian flying foxes (pteropid bats) (Field et al., 2001). Another henipaviral species, Cedar virus (CedV), was isolated from fruit bats in Australia (Marsh et al., 2012). HeV has a broad host range as it uses a highly conserved cellular protein, ephrin-B2, as an entry receptor (Marsh et al., 2012; Field, 2016). CedV also uses ephrin-B2 (Marsh et al., 2012). However, CedV and HeV use different coding strategy of the viral phosphoprotein (P) gene, which play fundamental role in antagonizing the innate immune system of the host (Marsh et al., 2012; Glennon et al., 2015). Unlike HeV, P gene of CedV lacks the coding capacity for the highly conserved V and W proteins (Figure 1A). V protein could be the major determinant of pathogenesis while W protein determines the disease course (Satterfield et al., 2015). When ferrets and guinea pigs were experimentally challenged with CedV, viral replication and the generation of neutralizing antibodies were observed, but no clinical disease was identified (Marsh et al., 2012).

Many bat-borne zoonotic viruses are highly pathogenic to spillover hosts, but bats are predominantly clinically asymptomatic and rarely display any signs of disease (Wynne et al., 2014). The mechanisms used by bats to balance the support and control of viral infections remain largely unknown. Transcriptomic profiling of viral infections is ideal for studying these mechanisms. We thus used RNA sequencing (RNA-seq) to identify differences in human and bat cell lines after two periods of infection with either HeV or CedV. Surprisingly, both cell lines exhibited a stronger immune response to CedV than to HeV. Our study provides novel insight into the host response to infection with phylogenetically similar, but pathogenically dissimilar viruses.

## Results

### Host Gene Transcription Following Infection With HeV or CedV

To explore the effects of HeV and CedV on host gene expression, we used RNA-seq to analyze HeV-, CedV-, or mock-infected immortalized cells lines from bats (PaKi) and humans (HeLa) 6 and 24 h post infection (hpi). Across all infection groups, the most differentially expressed genes (DEGs) were identified in CedV-infected PaKi cells at 24 hpi (4371 genes upregulated and 3896 downregulated, as compared to uninfected PaKi cells; Figure 1B). The fewest DEGs were identified in HeV-infected HeLa cells at 24 hpi (784 genes upregulated and 411 downregulated, as compared to uninfected HeLa cells). And more DEGs were identified in the CedV-infected cells than in HeV-infected cells, irrespective of cell type. Therefore, the host response to HeV and CedV differed, with CedV inducing more differential expression of host genes than HeV. In addition, more DEGs were identified in the bat cells than in the human cells, irrespective of infection type.

### Pattern Recognition Receptors (PRRs)

PRRs, which include toll-like receptors (TLRs) and retinoic acid inducible gene I (RIG-I) like receptors (RLRs), are vital to the host immune system as they form the first line of defense against infection (Baker et al., 2013). The RLR family comprises RIG-I, melanoma differentiation-associated protein 5 (MDA5), laboratory of genetics and physiology 2 (LGP2) (Satoh et al., 2010). RIG-I, MDA5, LGP2, and TLR3 were significantly upregulated in CedV-infected PaKi and HeLa cells at 24 hpi (Table 1). These genes were more strongly upregulated in PaKi cells than in HeLa cells.

**TABLE 1:** Log2 fold change (log2FC) of DEGs involved in pattern recognition in HeV- or CedV-infected PaKi and HeLa cells.

	PaKi				HeLa			
	HeV-6 hpi^a^	CedV-6 hpi^b^	HeV-24 hpi^c^	CedV-24 hpi^d^	HeV-6 hpi	CedV-6 hpi	HeV-24 hpi	CedV-24 hpi
RIG-I	–	–	–	7.79	–	0.62	–	5.23
MDA5	−0.49	−0.84	0.52	7.51	–	–	1.79	6.36
LGP2	–	–	–	9.68	–	–	–	3.99
TLR3	−0.95	−0.42	0.59	2.14	–	–	0.57	3.02
TLR4	–	–	–	–	0.42	0.83	–	0.36
TLR6	−0.47	-0.38	–	–	−0.30	-0.19	–	–
NLRC5	–	–	–	9.72	–	−0.59	–	0.75

**^*a*^HeV-infected corresponding PaKi or HeLa cell at 6 hpi. ^*b*^CedV-infected corresponding PaKi or HeLa cell at 6 hpi. ^*c*^HeV-infected corresponding PaKi or HeLa cell at 24 hpi. ^*d*^CedV-infected corresponding PaKi or HeLa cell at 24 hpi. ^*e*^The corresponding gene was not differentially expressed.**

NOD-like receptor family CARD domain containing 5 (NLRC5) is an important regulator of MHC class I gene expression (Ranjan et al., 2015). NLRC5 interacts with RIG-I to induce a robust response to the influenza virus; overexpression of NLRC5 resulted in impaired influenza viral replication (Ranjan et al., 2015). Here, NLRC5 was upregulated in CedV-infected PaKi and HeLa cells at 24 hpi, with more differential expression observed in PaKi cells. This indicated that NLRC5 may also interact with RIG-I to impair CedV replication. The presence of highly expressed PRRs in both CedV-infected cell lines suggested that both bats and humans have a strong antiviral response to CedV infection.

### Interferon (IFN) Response to Infection

**FIGURE 2:**
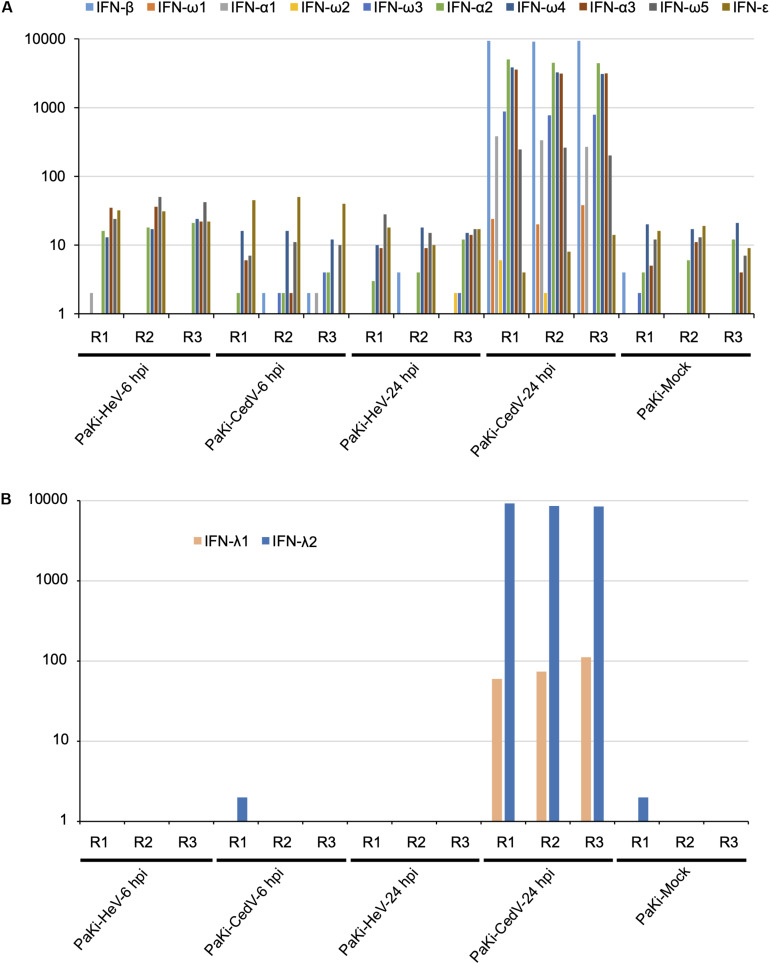
The transcriptional profiles of type I **(A)** and type III **(B)** interferon (IFN) in PaKi cells (HeV-, CedV-, or mock-infected at 6 or 24 hpi). The y-axes indicate the read counts per gene for type I and III IFN locus from *P. alecto*. R1, R2, and R3 represent the three biological replicates.

To determine the role of IFNs in the host response to viral infections, RNA-seq reads from PaKi cells (HeV-, CedV-, or mock-infected) were separately mapped to type I IFN locus (three IFN-α, one IFN-β, one IFN-ε, and five IFN-ω), using the type I IFN locus from *Pteropus alecto* as a reference (Zhou et al., 2016). IFN-α1, IFN-α2, IFN-α3, IFN-β, IFN-ω3, IFN-ω4, and IFN-ω5 were highly expressed in CedV-infected PaKi cells at 24 hpi, based on the read depth counts of the IFN transcripts, especially IFN-α2, IFN-α3, IFN-β, and IFN-ω4 (Figure 2). In contrast, type I IFN expression in PaKi cells showed little change following HeV infection. RNA-seq reads from PaKi cells were also mapped to the IFN-λ1 and IFN-λ2 genes of *P. Alecto* (Zhou et al., 2011). At 24 hpi with CedV, IFN-λ2 expression in PaKi cells was high, while IFN-λ1 expression was relatively low. Consistent with our results for type I IFN genes, IFN-λ1 and IFN-λ2 expression did not increase in HeV-infected PaKi cells. HeV-, CedV-, or Mock-infected HeLa cells were also mapped against human genome. In CedV-infected HeLa cells, IFN-λ (λ1, λ2, and λ3) and IFN-β were both upregulated at 24 hpi, but no IFN genes were differentially expressed in HeV-infected HeLa cells (Table 1).

IFNs stimulate antiviral activity by inducing interferon-stimulated genes (ISGs), including the IFN-induced protein with the tetratricopeptide repeats (IFIT) (Fensterl and Sen, 2015). We examined the expression of IFITs during HeV and CedV infection. IFIT1, IFIT2, IFIT3, and IFIT5 were significantly upregulated in CedV-infected HeLa and PaKi cells (Table 2). Indeed, the expression of IFIT1, IFIT2, and IFIT3 increased over 2000-fold in the CedV-infected PaKi cells, as compared to uninfected PaKi cells. Unexpectedly, IFIT1, IFIT2, and IFIT3 were also upregulated in HeV-infected HeLa cells at 24 hpi.

**TABLE 2:** Log2FC of the DEGs involved in the IFN response to HeV and CedV infection in PaKi and HeLa cells.

	PaKi				HeLa			
	HeV-6 hpi^a^	CedV-6 hpi^b^	HeV-24 hpi^c^	CedV-24 hpi^d^	HeV-6 hpi	CedV-6 hpi	HeV-24 hpi	CedV-24 hpi
IFN-β	N^e^	N	N	N	–^f^	–	–	13.92
IFN-λ1	N	N	N	N	–	–	–	13.36
IFN-λ2	N	N	N	N	–	–	–	10.68
IFN-λ3	N	N	N	N	–	–	–	10.88
IFIT1	–	–	–	11.90	−0.85	−1.50	1.06	5.91
IFIT2	–	–	–	11.96	–	–	1.46	6.53
IFIT3	–	–	–	11.00	0.36	0.60	1.23	5.86
IFIT5	–	−0.81	–	1.86	–	0.51	–	2.85
OAS1	–	–	–	–	–	–	–	3.71
OAS2	–	–	–	8.46	–	–	–	9.31
OAS3	–	−0.62	0.52	5.85	0.54	0.24	0.53	3.66
OASL	–	–	–	11.42	–	–	1.18	5.22
IRF3	–	–	–	0.93	–	–	–	–

**^*a*^HeV-infected corresponding PaKi or HeLa cell at 6 hpi. ^*b*^CedV-infected corresponding PaKi or HeLa cell at 6 hpi. ^*c*^HeV-infected corresponding PaKi or HeLa cell at 24 hpi. ^*d*^CedV-infected corresponding PaKi or HeLa cell at 24 hpi. ^*e*^The expression of corresponding gene was measured based on the read depth counts, and Log2FC of was not calculated. ^*f*^The corresponding gene was not differentially expressed.**

The 2′,5′-oligoadenylate synthetase (OAS) proteins can be induced by type I IFNs (Sadler and Williams, 2008). Consistent with this, OAS2, OAS3, and OASL were significantly upregulated in CedV-infected HeLa and PaKi cells at 24 hpi, with OAS1 upregulated in CedV-infected PaKi cells at 24 hpi. The HeV V and W proteins block IFN production, with V inhibiting MDA5 signaling and W blocking IFN synthesis from TLRs after IFN regulatory factor 3 (IRF3) activation (Glennon et al., 2015). At 24 hpi, MDA5 and TLRs were upregulated in CedV-infected PaKi and HeLa cells, while IRF3 was upregulated in CedV-infected PaKi cells only (Table 2).

### Apoptosis

**FIGURE 3:**
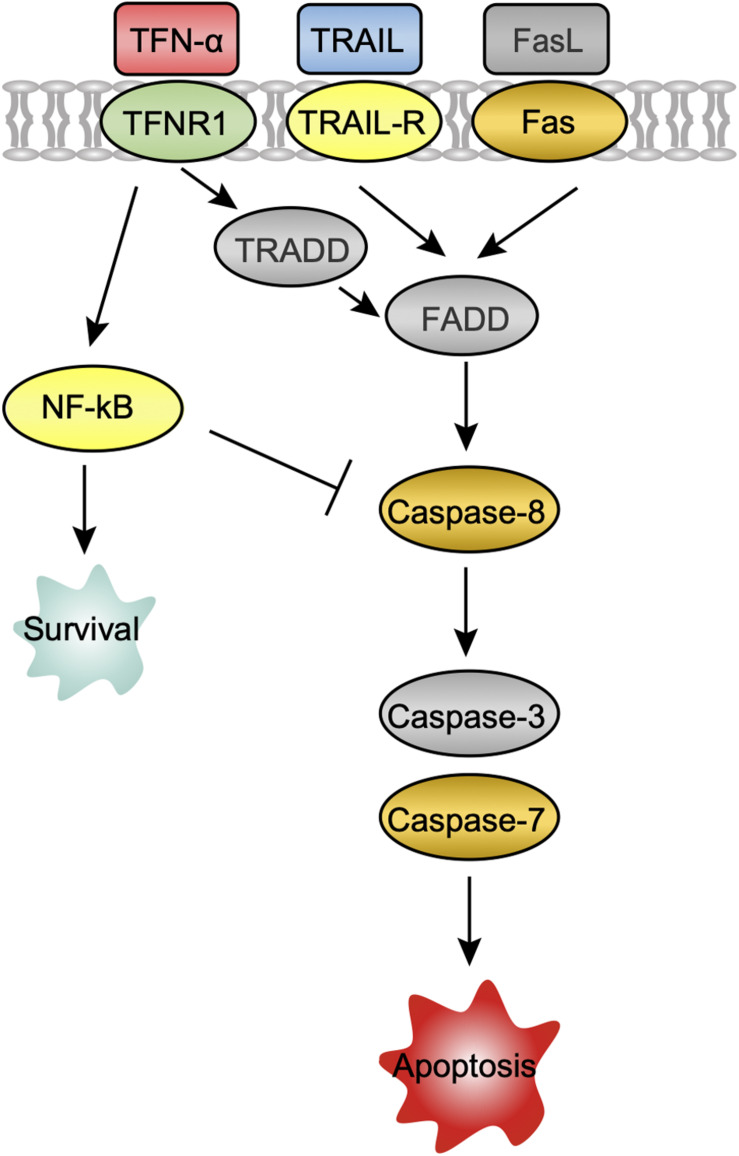
A potential apoptosis pathway in PaKi cells following CedV infection. The challenge groups in which genes were upregulated at 24 hpi are indicated with color. Red: CedV-infected PaKi cells only; Green: CedV-infected HeLa and PaKi cells; Blue: HeV- or CedV-infected PaKi cells; Orange: HeV- or CedV-infected PaKi cells and CedV-infected HeLa cells; Yellow: HeV- or CedV-infected HeLa and PaKi cells. Genes that were not upregulated in any challenge group at 24 hpi are colored gray.

Several host mechanisms that prevent viral replication, viral dissemination, or persistent viral infection involve programmed cell death, or apoptosis (Barber, 2001). The extrinsic apoptosis pathway is triggered by the binding of death receptors (TNF receptor 1 (TNFR1), TNF-related apoptosis-inducing ligand (TRAIL) receptor 1/2 (TNFRSF10A/B), and FasL-related receptor Fas to their respective ligands (TNF-α, TRAIL, and FasL) (Cuda et al., 2016). At 24 hpi, the ligands TNF-α and TRAIL, death receptors TNFR1, TNFRSF10B, Fas, as well as apoptosis signaling protein DAXX were upregulated in CedV-infected PaKi cells, suggesting that TNF-α-, TRAIL-, and FasL-mediated apoptosis may be induced (Figure 3). And the expression of TNF-α, TRAIL, TNFRSF10B, and DAXX in CedV-infected PaKi increased more than 3-fold over uninfected PaKi cells (Table 3). According to Table 3, bat induced a relatively stronger apoptosis response at 24 hpi than at 6 hpi. However, at 24 hpi, in CedV-infected HeLa cells, TNFR1, TNFRSF10A, TNFRSF10B and Fas were only slightly upregulated as compared to uninfected HeLa cells. In addition, few genes were upregulated in HeV-infected PaKi and HeLa cells at 6 and 24 hpi.

**TABLE 3:** Log2FC of DEGs involved in apoptosis in HeV- or CedV-infected PaKi and HeLa cells.

	PaKi				HeLa			
	HeV-6 hpi^a^	CedV-6 hpi^b^	HeV-24 hpi^c^	CedV-24 hpi^d^	HeV-6 hpi	CedV-6 hpi	HeV-24 hpi	CedV-24 hpi
TNF-α	–^e^	–	–	12.41	–	–	–	–
TRAIL	−1.25	−3.28	0.86	3.47	–	–	–	–
TNFR1	–	0.84	–	0.54	–	–	–	0.38
TNFRSF10A	–	–	–	–	0.74	1.01	–	0.37
TNFRSF10B	1.66	2.52	0.93	3.31	0.45	0.78	0.29	0.32
FAS	–	0.24	0.44	0.95	–	0.54	–	0.76
FADD	–	–	–	−0.30	–	–	–	–
DAXX	0.45	0.45	–	2.29	–	–	–	–
TRADD	–	–	–	–	–	−0.56	–	–
CASP8	–	–	0.45	1.70	–	–	–	0.62
CASP3	–	−0.23	–	−1.04	0.31	0.43	–	–
CASP7	0.93	−0.82	0.31	0.64	0.45	0.63	–	0.93
RIP1	–	–	–	1.11	–	0.28	–	0.33
TRAF2	0.49	0.65	–	1.36	0.61	0.71	–	–
AIP	−0.37	−0.53	–	−0.99	–	0.37	–	–
ASK1	–	−0.62	–	1.47	0.29	–	–	–
JNK1	–	0.95	–	0.27	–	0.39	–	–
JNK2	–	−0.44	–	−0.82	–	–	–	–
JUN	–	−0.34	–	2.11	0.62	0.88	–	0.53
AP1	–	1.23	–	4.01	−0.76	0.38	0.67	0.68
p53	0.37	–	0.42	0.59	−0.95	−0.95	0.32	0.40
Bim	–	1.47	–	1.34	–	0.77	–	0.27

**^*a*^HeV-infected corresponding PaKi or HeLa cell at 6 hpi. ^*b*^CedV-infected corresponding PaKi or HeLa cell at 6 hpi. ^*c*^HeV-infected corresponding PaKi or HeLa cell at 24 hpi. ^*d*^CedV-infected corresponding PaKi or HeLa cell at 24 hpi. ^*e*^The corresponding gene was not differentially expressed.**

Caspase-8 was only upregulated in CedV-infected PaKi and HeLa cells, as well as in HeV-infected PaKi cells at 24 hpi. There was an over 2-fold increase in the expression of caspase-8 in CedV-infected PaKi cells, as compared to uninfected cells. It is therefore probable that CedV sensitizes human and bat cells to TNF-α-, TRAIL-, and FasL-mediated apoptosis more effectively than does HeV (Figure 3). The overexpression of caspase-7 indicated that this protein may be also involved in apoptosis-mediated cell death. Caspase-3 was only upregulated in HeV-infected HeLa cells (Table 3). At 24 hpi, another pro-apoptotic group of genes (DAXX, RIP1, TRAF2, ASK1, JUN, AP1, and Bim) were induced by over 2-fold in CedV-infected PaKi cells, implying that PaKi cells were also subjected to strong pro-apoptotic signals.

### Cytokines

**FIGURE 4:**
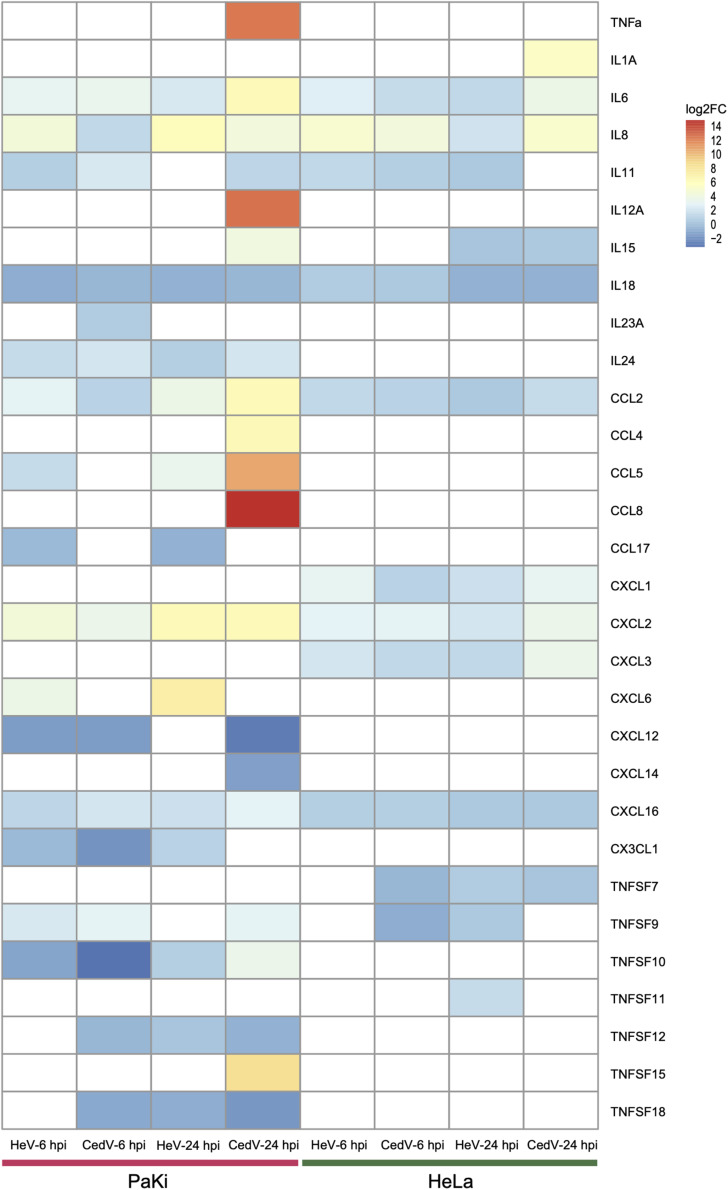
Cytokine expression in HeV- or CedV-infected HeLa and PaKi cells at 6 or 24 hpi. The color scale for log2FC is shown at the top right of the figure. Genes that were not differentially expressed are colored white. HeV-6 hpi and CedV-6 hpi represent HeV- or CedV-infected corresponding PaKi or HeLa at 6 hpi. HeV-24 hpi and CedV-24 hpi represent HeV- or CedV-infected corresponding PaKi or HeLa at 24 hpi.

In response to viral infection, target cells produce cytokines and chemokines to control viral replication (Klotman and Chang, 2006). Here, the cytokines IL6, IL8, CCL2, CXCL2, and CXCL16 were upregulated in HeV- or CedV-infected PaKi and HeLa cells at 6 and 24 hpi, suggesting that cytokines may function during HeV or CedV infection in PaKi and HeLa cells. It is notable that more cytokines were significantly upregulated in CedV-infected PaKi cells than in HeV-infected PaKi cells or in HeLa cells infected with either virus at 24 hpi (Figure 4). In particular, the expression levels of TNF-α, IL12A, CCL5, and CCL8 increased over 1000-fold in CedV-infected PaKi cells, as compared to uninfected cells. The large number of highly expressed cytokines in CedV-infected PaKi cells suggested that the bat immune system responds strongly to CedV infection.

### NF-κB Signaling

The nuclear factor-κB (NF-κB) family of transcription factors regulate innate and acquired host immune responses, and is thus critical for the host response to microbial pathogen infection (Rahman and McFadden, 2011). The mammalian NF-κB proteins are members of the Rel domain-containing protein family: RELA, RELB, c-REL, the NF-κB p105 subunit (NF-κB1), and the NF-κB p100 subunit (NF-κB2). The expression levels of RELA, RELB, c-REL, NF-κB1, NF-κB2, and NF-κB inhibitor-α (IκBα) were affected by HeV and CedV infection in both PaKi and HeLa cells at 6 and 24 hpi, but the majority of these genes were most highly expressed in CedV-infected PaKi cells at 24 hpi (Supplementary Table S1). Both TRAF2 and RIP1, which interact with TNFR1 in the classical NF-κB pathway, were highly expressed in CedV-infected PaKi cells at 24 hpi (Table 3), suggesting that TNF-α is involved in the activation of NF-κB, leading to the transcription of genes that encode pro-inflammatory and proliferative factors. Thus, both HeV and CedV might induce an immune response in PaKi and HeLa cells via NF-κB signaling. The immune response was strongest in CedV-infected PaKi cells.

### JAK–STAT Signaling

The cytokine-activated Janus kinase (JAK)–signal transducer and activator of transcription (STAT) pathway play an important role in the control of immune responses (Shuai and Liu, 2003). Here, JAK2, JAK3, STAT1, STAT2, and STAT3 were over 2-fold upregulated in CedV-infected PaKi cells at 24 hpi. In contrast, at 24 hpi, JAKs and STATs were expressed at relatively low levels in CedV-infected HeLa cells. The expression levels of JAKs and STATs in HeLa and PaKi cells showed little change following HeV infection (Supplementary Table S2) at 6 and 24 hpi, indicating that CedV infection stimulated JAK-STAT signaling but HeV infection might not. Interestingly, the suppressor of cytokine signaling (SOCS) proteins that regulate the JAK-STAT were also upregulated in CedV-infected PaKi cells at 24 hpi (Supplementary Table S2). As SOCS inhibits JAK-STAT signaling (Shuai and Liu, 2003), its upregulation indicates a classic negative-feedback loop.

### Genes Highly Differentially Expressed Following HeV and CedV Infection

**FIGURE 5:**
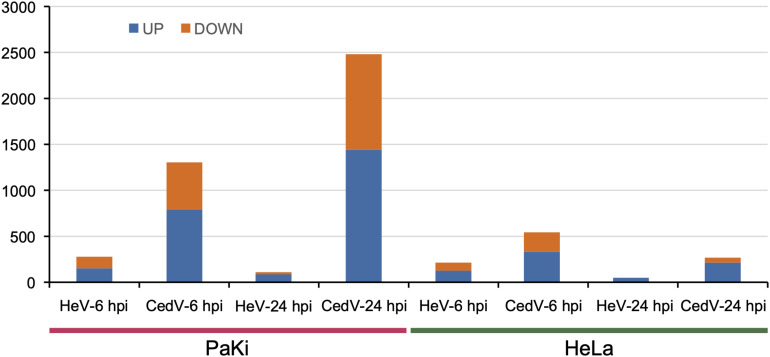
Genes highly up- and downregulated (>2-fold change in expression as compared to uninfected cells) in HeV- or CedV-infected HeLa and PaKi cells at 6 or 24 hpi. HeV-6 hpi and CedV-6 hpi represent HeV- or CedV-infected corresponding PaKi or HeLa at 6 hpi. HeV-24 hpi and CedV-24 hpi represent HeV- or CedV-infected corresponding PaKi or HeLa at 24 hpi.

To better understand the functions of genes strongly up- and downregulated post infection, we selected DEGs with > 2-fold changes in expression level in both cell lines after HeV or CedV infection, as compared to uninfected cells. At 24 hpi, CedV-infected PaKi cells had the most DEGs with a > 2-fold change in expression (1441 DEGs upregulated and 1039 downregulated) (Figure 5). At 24 hpi, the fewest DEGs with a > 2-fold change in expression were observed in HeV-infected HeLa cells (46 upregulated and 4 downregulated).

We then used Kyoto encyclopedia of genes and genomes (KEGG) pathway enrichment analysis to predict potential interactions among these selected DEGs. Our KEGG pathway analysis indicated that for DEGs with a > 2-fold upregulation the most frequently predicted pathways were the TNF signaling pathway, the NF-κB signaling pathway, and the NOD-like receptor signaling pathway (Supplementary Table S3). Notably, all genes significantly upregulated at 6 or 24 hpi in all challenge groups were enriched in the TNF signaling pathway. In addition, selected DEGs upregulated in the HeV-infected PaKi cells, HeV-infected HeLa cells, and CedV-infected HeLa cells were enriched in the NF-κB signaling pathway at 24 hpi. However, CedV-infected PaKi cells were not highly enriched in the NF-κB pathway at 24 hpi. We did not perform a KEGG pathway analysis on the selected DEGs downregulated in the HeV-infected HeLa cells at 24 hpi, as only four genes were highly downregulated. In contrast to the upregulated genes, distinct pathways were enriched in the downregulated DEGs, and there were no shared pathways across the challenge groups (Supplementary Table S4).

## Discussion

Here, we used the expression profiles of PaKi and HeLa cells infected with HeV or CedV to identify differences in host response to these henipaviruses. We found that host responses to HeV and CedV infection differed dramatically. The large number of DEGs in CedV-infected PaKi cells at 24 hpi indicated that the bat response to CedV infection was strong. In contrast, relatively few DEGs were identified in HeV-infected HeLa cells at 24 hpi. Various immune related proteins (e.g., PRRs, IFNs, and cytokines) and pathways (e.g., apoptosis, NF-κB signaling, and JAK–STAT signaling) were upregulated in PaKi and HeLa cells following CedV infection. The immune response was relatively stronger in PaKi cells than in HeLa cells. One possible explanation for this discrepancy is that the CedV P gene does not encode V and W proteins; these proteins may antagonize host innate immunity, including blocking IFN production (Glennon et al., 2015). Because our results indicated that CedV infection induces interferon expression and other immune signaling pathways in both HeLa and PaKi cells, it is probable that V and W proteins affect several pathways related to immunity.

Previous study showed that no clinical disease was observed when Cedar virus was tested in experimental challenge models in ferrets and guinea pigs (Marsh et al., 2012). One explanation for this is that ferrets and guinea pigs may control viral replication as part of an innate antiviral response, such as PRRs, IFNs, cytokines, and apoptosis-related factors. The immune responses of HeLa and PaKi cells to HeV infection were weak, possibly because V and W proteins were present. This is consistent with a recent study showing that V and W proteins were crucial for pathogenesis and disease progression, respectively (Satterfield et al., 2015).

The greater production of cytokines by CedV-infected PaKi cells compared to HeV-infected PaKi cells observed here was likely due to the absence of the W protein in CedV, as it has been shown that cells infected with W-deficient NiV produce more cytokines than in cells infected with wild-type NiV (Satterfield et al., 2015). The W protein may control the cytokine response of target endothelial cells, thus affecting disease progression without altering disease lethality (Satterfield et al., 2015). Therefore, although cytokine production may not be directly responsible for the distinct clinical outcomes of HeV and CedV infections, cytokine production may affect disease progression.

Several genes upregulated in HeV-infected HeLa and PaKi cells encoded cytokines or elements of the NF-κB signaling pathway. Moreover, our KEGG pathway analysis of highly upregulated DEGs indicated that the TNF and NF-κB signaling pathways were enriched in HeV-infected HeLa and PaKi cells, suggesting that HeV-infection induced certain innate antiviral responses in both cell lines. Several proteins crucial to apoptosis, such as TRAIL, TNFRSF10B, FAS, CASP7, and CASP8, were upregulated in HeV-infected PaKi cells at 24 hpi, suggesting that HeV infection may trigger apoptosis in PaKi cells. This is consistent with a previous study, which indicated that HeV induces apoptosis in bat cells, but not human cells (Wynne et al., 2014). Both HeV and CedV could induce apoptosis in bat, which implied that apoptosis pathway may contribute to control the viral infection in bat. However, apoptosis could not be induced by HeV in HeLa cells and the immune response is extremely weak compared to HeLa cells infected with CedV, thus may result in divergent clinical outcomes of human infected with HeV or CedV. A comparative analysis based on the relative codon deoptimization index (RCDI) for host adaptation of HeV, CedV, Nipah virus, and Hendra like Mojiang virus revealed that except for dog and hamster, all other evaluated hosts (human, bat, horse, pig, cat, ferret, squirrel monkey, and African green monkey) were most susceptible to HeV while all hosts were least susceptible to CedV (Khandia et al., 2019), which may also provide insight to the distinct clinical outcomes of HeV and CedV. At 24 hpi, the expression levels of IFIT1, IFIT2, and IFIT3 were over 2-fold up-regulated in HeV-infected HeLa cells (Table 2), probably because genes such as IRF7 can induce the expression of ISGs in the absence of type I or III IFN (Schmid et al., 2010). In PaKi cells, there were several DEGs (e.g., MDA5, TLR3, IFIT5, TRAIL, and JUN) downregulated at 6 hpi but significantly upregulated at 24 hpi, especially infected with CedV. In general, the host response to viral infection is stronger at 24 hpi than that at 6 hpi. Probably 24 h post infection could be more appropriate to analyze the host response.

We observed several points of similarity between the two viruses. For example, some DNA damage checkpoint and innate immune genes shown to be under positive selection in bats (e.g., p53, c-REL, and RAD50) (Zhang et al., 2013) were upregulated in PaKi cells infected with either virus, suggesting that the certain innate antiviral mechanisms activated by henipaviruses are conserved across bat lineages. In summary, we used RNA-seq to compare bat and human cell lines infected with HeV or CedV. We found that CedV caused a stronger innate immune response than did HeV. It is possible that differences in the phosphoprotein gene coding strategy between the two viruses lead to host transcriptomic divergence and alterations in viral lethality. Further work is required to understand how henipaviruses evade the host immune response.

## Materials and Methods

### Cell Culture

The *P. alecto* immortalized kidney-derived PaKi cell line (Crameri et al., 2009) and the human HeLa cell line were used in this study. Cell lines were grown in either Dulbecco’s modified Eagle’s medium (DMEM)/F12 (PaKiT03) or DMEM GlutaMAX (HeLa), both supplemented with 10% (v/v) fetal calf serum (FCS) and 100 U/mL penicillin/streptomycin. All cells were incubated at 37°C in a humidified atmosphere containing 5% CO_2_.

### HeV and CedV Infection of Bat and Human Cell Lines

All virology work was conducted at the BSL-4 facility of the CSIRO Australian Animal Health Laboratory (Geelong, Australia). Approximately 2 × 10^7^ PaKi and HeLa cells per challenge were mock infected or infected with HeV (Hendra virus/Australia/horse/1994/Hendra) or CedV (Cedar virus/Australia/bat/2009/Cedar) for 6 or 24 h at a MOI of 10. We performed three biological replicates for each challenge in T75cm^2^ flasks. All cells were harvested with trypsinization and resuspended in RLT buffer (Qiagen, Hilden, Germany). Mock-infected cells were used as controls.

### RNA Isolation, Sequencing, and RNA-Seq Analysis

Total RNA was isolated using the Qiagen RNeasy Mini kit (Qiagen, Hilden, Germany) and treated with DNase I (Qiagen, Hilden, Germany). The quality and quantity of total RNA was assessed using a Bioanalyzer (Agilent, Santa Carla, CA, United States). mRNA was sequenced on an Illumina HiSeq 2000 sequencer (Illumina, San Diego, CA, United States). Adapter sequences were trimmed from the resulting reads. The quality of reads was assessed using FastQC^1^. High-quality reads were mapped to either the *P. alecto* or human genomes using TopHat (version 2.1.1) (Trapnell et al., 2009). Based on the resulting alignments, we assembled our transcripts separately by challenge group. Grouped transcripts were assembled and merged using Cufflinks and Cuffmerge (version 2.2.1) (Trapnell et al., 2012). Differential expression analysis was performed with Cuffdiff (version 2.2.1) (Trapnell et al., 2012). Transcripts with a fragments per kilobase of transcript per million mapped reads (FPKM) value < 1 before or after HeV or CedV infection were discarded. We considered a transcript statistically significant if the FDR-adjusted *p*-value of the test statistic (*q*-value) was < 0.05.

### Analysis of IFN Expression

As IFN genes are only partially characterized in the *P. alecto* genome, PaKi RNA-seq reads were mapped separately to the *P. alecto* type I IFN locus (Zhou et al., 2016), IFN-λ1 (GenBank accession no. HQ201956.1) and IFN-λ2 (GenBank accession no. HQ201955.1) using bowtie2 (Langmead and Salzberg, 2012). We used these mappings to measure the expression of type I and III IFNs. We used SAMtools to compile and count the number of reads mapped (Li et al., 2009).

### Functional Enrichment Analysis

To predict the molecular interactions of the DEGs, we performed KEGG enrichment analysis with KOBAS (Xie et al., 2011), using a hypergeometric test. We considered KEGG pathways with *p* < 0.05 significantly enriched. The -log10 (*p*-value) indicates the enrichment score, which represents the significance of the corresponding pathway enrichment.

## Data Availability Statement

The original contributions presented in the study are publicly available. This data can be found here: https://bigd.big.ac.cn/gsa, accession number CRA002578.

## Author Contributions

L-FW and JC designed the research and supervised the experiments. JC, MT, and GM performed the experiments. JC and MC analyzed the data. JC, MC, and L-FW wrote the manuscript.

## Conflict of Interest

The authors declare that the research was conducted in the absence of any commercial or financial relationships that could be construed as a potential conflict of interest.

## Footnotes

1 http://www.bioinformatics.babraham.ac.uk/projects/fastqc/
