# Supplementary material for: Distinct Cell Transcriptomic Landscapes Upon Henipavirus Infections
Source: Front Microbiol. 2020 May 19;11:986. doi: 10.3389/fmicb.2020.00986 (PMC7248276; doi:10.3389/fmicb.2020.00986)
Supplement: Supplementary file 1 [file Data_Sheet_1.zip › Supplementary Table 1.DOCX]

Supplementary Table S1 Log2FC of DEGs involved in NF-κB signaling in HeV- or CedV-infected PaKi and HeLa cells

|  | PaKi | | | |  | HeLa | | | |
| --- | --- | --- | --- | --- | --- | --- | --- | --- | --- |
|  | HeV-  6 hpi^a^ | CedV-  6 hpi^b^ | HeV-  24 hpi^c^ | CedV-24 hpi^d^ |  | HeV-  6 hpi | CedV-  6 hpi | HeV-  24 hpi | CedV-24 hpi |
| RELA | 0.39 | 0.63 | -^e^ | 1.00 |  | 0.40 | 0.81 | - | - |
| RELB | 1.22 | 3.14 | 1.47 | 4.55 |  | 2.98 | 2.84 | 1.11 | 1.64 |
| c-REL | - | 1.32 | 0.94 | 3.97 |  | 0.43 | - | 0.42 | 0.38 |
| NF-κB1 | 1.00 | 2.37 | 0.94 | 3.26 |  | 1.06 | 1.60 | - | 0.66 |
| NF-κB2 | 0.72 | 1.27 | 0.86 | 2.53 |  | 2.10 | 2.03 | 0.71 | 1.00 |
| IκBα | 1.74 | 1.41 | 2.50 | 4.48 |  | 1.24 | 1.04 | 0.60 | 1.59 |
| NIK | - | 0.63 | - | 2.29 |  | 0.24 | 0.46 | -0.36 | - |
| IKKα | - | - | - | 0.33 |  | 0.27 | 0.34 | - | - |
| IKKβ | - | -0.44 | 0.41 | 0.64 |  | - | - | - | - |
| NEMO | - | 0.64 | - | -0.38 |  | - | - | - | - |

^a^ HeV-infected corresponding PaKi or HeLa cell at 6 hpi.

^b^ CedV-infected corresponding PaKi or HeLa cell at 6 hpi.

^c^ HeV-infected corresponding PaKi or HeLa cell at 24 hpi.

^d^ CedV-infected corresponding PaKi or HeLa cell at 24 hpi.

^e^ The corresponding gene was not differentially expressed.
