# Supplementary material for: Distinct Cell Transcriptomic Landscapes Upon Henipavirus Infections
Source: Front Microbiol. 2020 May 19;11:986. doi: 10.3389/fmicb.2020.00986 (PMC7248276; doi:10.3389/fmicb.2020.00986)
Supplement: Supplementary file 1 [file Data_Sheet_1.zip › Supplementary Table 2.DOCX]

Supplementary Table S2 Log2FC of DEGs involved in JAK-STAT signaling in HeV- or CedV-infected PaKi and HeLa cells

|  | PaKi | | | |  | HeLa | | | |
| --- | --- | --- | --- | --- | --- | --- | --- | --- | --- |
|  | HeV-  6 hpi^a^ | CedV-  6 hpi^b^ | HeV-  24 hpi^c^ | CedV-24 hpi^d^ |  | HeV-  6 hpi | CedV-  6 hpi | HeV-24 hpi | CedV-24 hpi |
| JAK1 | -^e^ | - | - | - |  | 0.83 | 0.75 | - | 0.27 |
| JAK2 | - | - | 0.46 | 3.00 |  | 0.48 | 0.53 | - | 0.78 |
| JAK3 | - | - | - | 3.79 |  | - | - | - | - |
| TYK2 | - | -0.23 | - | - |  | -0.33 | -0.34 | - | - |
| STAT1 | - | -0.32 | - | 3.19 |  | 0.28 | - | - | 2.55 |
| STAT2 | - | -0.43 | - | 2.69 |  | -0.75 | -0.69 | - | 2.17 |
| STAT3 | - | - | - | 1.64 |  | - | 0.42 | - | 0.26 |
| STAT5A | - | -0.60 | 0.30 | 0.99 |  | 1.04 | 1.07 | 0.25 | 0.54 |
| STAT6 | - | - | 0.36 | 0.81 |  | - | - | - | - |
| SOCS1 | - | - | - | 7.49 |  | - | 1.65 | - | 2.57 |
| SOCS2 | -0.87 | - | -0.42 | 1.67 |  | - | - | - | - |
| SOCS3 | 0.67 | 2.46 | 0.31 | 2.37 |  | - | 0.27 | 0.29 | 0.40 |
| SOCS4 | - | 0.73 | - | 0.27 |  | - | 0.21 | - | -0.32 |
| SOCS5 | - | 0.54 | - | 1.04 |  | - | - | - | - |
| SOCS6 | - | 1.14 | - | 0.53 |  | - | 0.26 | - | - |
| SOCS7 | - | 0.82 | - | 0.72 |  | - | - | - | - |

^a^ HeV-infected corresponding PaKi or HeLa cell at 6 hpi.

^b^ CedV-infected corresponding PaKi or HeLa cell at 6 hpi.

^c^ HeV-infected corresponding PaKi or HeLa cell at 24 hpi.

^d^ CedV-infected corresponding PaKi or HeLa cell at 24 hpi.

^e^ The corresponding gene was not differentially expressed.
