# Supplementary material for: Distinct Cell Transcriptomic Landscapes Upon Henipavirus Infections
Source: Front Microbiol. 2020 May 19;11:986. doi: 10.3389/fmicb.2020.00986 (PMC7248276; doi:10.3389/fmicb.2020.00986)
Supplement: Supplementary file 1 [file Data_Sheet_1.zip › Supplementary Table 3.DOCX]

Supplementary Table S3 KEGG pathway enrichment analysis of DEGs with > 2-fold upregulation in HeV- or CedV-infected HeLa and PaKi cells at 6 or 24 hpi, as compared to uninfected cells.

|  | **-log10 (p-value)^a^** | | | | | | | | |
| --- | --- | --- | --- | --- | --- | --- | --- | --- | --- |
|  | **PaKi** | | | |  | **HeLa** | | | |
|  | **HeV-**  **6 hpi^b^** | **CedV-**  **6 hpi^c^** | **HeV-**  **24 hpi^d^** | **CedV-**  **24 hpi^e^** |  | **HeV-**  **6 hpi** | **CedV-**  **6 hpi** | **HeV-**  **24 hpi** | **CedV-**  **24 hpi** |
| TNF signaling pathway | 10.99 | 9.54 | 14.16 | 11.70 |  | 19.05 | 12.79 | 10.11 | 10.26 |
| NF-kB signaling pathway | 7.14 | 6.74 | 7.92 | -^f^ |  | 13.46 | 8.94 | 8.76 | 10.98 |
| NOD-like receptor signaling pathway | 7.65 | - | 9.57 | - |  | 12.13 | 7.18 | 6.11 | 10.10 |
| Legionellosis | - | - | 6.36 | - |  | 13.97 | 7.29 | 10.05 | 11.70 |
| HTLV-I infection | 6.66 | 4.59 | - | 7.79 |  | 7.80 | 7.10 | - | - |
| Influenza A | 7.19 | - | - | 11.55 |  | 6.86 | - | - | 27.59 |
| Toll-like receptor signaling pathway | 6.98 | - | 7.77 | 7.40 |  | - | - | - | - |
| AGE-RAGE signaling pathway in diabetic complications | 6.60 | - | - | - |  | 10.08 | 9.71 | - | - |
| Transcriptional misregulation in cancer | 6.05 | 5.03 | - | 8.09 |  | - | - | - | - |
| Cytokine-cytokine receptor interaction | - | - | - | - |  | 7.71 | - | 6.15 | 12.61 |
| Osteoclast differentiation | 6.04 | 3.01 | - | - |  | - | 7.47 | - | - |
| Chagas disease (American trypanosomiasis) | 6.56 | - | 7.39 | - |  | - | - | - | - |
| Pertussis | - | - | 8.64 | - |  | - | - | 7.43 | - |
| Chemokine signaling pathway | - | - | 6.79 | - |  | - | - | - | - |
| Malaria | - | - | 6.76 | - |  | - | - | - | - |
| Rheumatoid arthritis | - | - | 6.68 | - |  | - | - | 7.03 | - |
| Ribosome biogenesis in eukaryotes | - | 5.49 | - | - |  | - | - | - | - |
| Apoptosis | - | 4.42 | - | 7.26 |  | - | - | - | - |
| MAPK signaling pathway | - | 3.24 | - | - |  | - | 7.18 | - | - |
| p53 signaling pathway | - | 2.99 | - | - |  | - | - | - | - |
| Hepatitis B | - | 2.99 | - | - |  | - | - | - | 10.22 |
| Herpes simplex infection | - | - | - | 13.67 |  | - | - | - | 20.12 |
| RIG-I-like receptor signaling pathway | - | - | - | 7.72 |  | - | - | - | - |
| Measles | - | - | - | 7.59 |  | - | - | - | 18.43 |
| Hepatitis C | - | - | - | 7.25 |  | - | - | - | 17.20 |
| Pathways in cancer | - | - | - | - |  | 13.65 | 9.38 | - | - |
| Epstein-Barr virus infection | - | - | - | - |  | 7.54 | - | - | - |
| Salmonella infection | - | - | - | - |  | - | - | 7.15 | - |
| Staphylococcus aureus infection | - | - | - | - |  | - | - | 6.11 | - |
| Complement and coagulation cascades | - | - | - | - |  | - | - | 5.57 | - |
| PI3K-Akt signaling pathway | - | - | - | - |  | - | 6.44 | - | - |

^a^ The significance of the corresponding pathway enrichment. For each infection group, only KEGG terms within top ten enrichment scores are presented.

^b^ HeV-infected corresponding PaKi or HeLa cell at 6 hpi.

^c^ CedV-infected corresponding PaKi or HeLa cell at 6 hpi.

^d^ HeV-infected corresponding PaKi or HeLa cell at 24 hpi.

^e^ CedV-infected corresponding PaKi or HeLa cell at 24 hpi.

^f^ KEGG term was not enriched in corresponding cells.
