# Supplementary material for: Distinct Cell Transcriptomic Landscapes Upon Henipavirus Infections
Source: Front Microbiol. 2020 May 19;11:986. doi: 10.3389/fmicb.2020.00986 (PMC7248276; doi:10.3389/fmicb.2020.00986)
Supplement: Supplementary file 1 [file Data_Sheet_1.zip › Supplementary Table 4.DOCX]

Supplementary Table S4 KEGG pathway enrichment analysis of DEGs with > 2-fold downregulation in HeV- or CedV-infected HeLa and PaKi cells at 6 or 24 hpi, as compared to uninfected cells.

|  | **-log10 (p-value)^a^** | | | | | | | |
| --- | --- | --- | --- | --- | --- | --- | --- | --- |
|  | **PaKi** | | | |  | **HeLa** | | |
|  | **HeV-6 hpi^b^** | **CedV-6 hpi^c^** | **HeV-24 hpi^d^** | **CedV-24 hpi^e^** |  | **HeV-6 hpi** | **CedV-6 hpi** | **CedV-24 hpi** |
| Pathways in cancer | 2.91 | 2.24 | -^f^ | 5.11 |  | - | - | 3.67 |
| ABC transporters | 2.38 | - | - | - |  | - | - | - |
| Neuroactive ligand-receptor interaction | 2.02 | - | 2.33 | - |  | 3.42 | - | - |
| Bile secretion | 1.95 | - | - | - |  | - | - | - |
| FoxO signaling pathway | 1.92 | 2.40 | - | - |  | - | - | - |
| Rap1 signaling pathway | 1.88 | - | - | - |  | 2.91 | - | - |
| Vascular smooth muscle contraction | 1.41 | 1.89 | - | - |  | - | - | - |
| Malaria | 1.36 | - | - | - |  | - | - | - |
| Leukocyte transendothelial migration | 1.35 | - | - | - |  | - | - | - |
| Cardiac muscle contraction | - | - | 3.88 | - |  | - | - | - |
| Adrenergic signaling in cardiomyocytes | - | - | 3.09 | - |  | - | - | - |
| Hypertrophic cardiomyopathy (HCM) | - | - | 2.33 | 4.53 |  | - | - | - |
| Dilated cardiomyopathy | - | - | 2.28 | 4.78 |  | - | - | - |
| Wnt signaling pathway | - | 2.31 | 1.89 | - |  | - | - | - |
| cGMP-PKG signaling pathway | - | - | 1.74 | - |  | - | - | - |
| Terpenoid backbone biosynthesis | - | - | 1.59 | - |  | - | - | - |
| Steroid biosynthesis | - | - | 1.57 | - |  | - | - | - |
| Proximal tubule bicarbonate reclamation | - | - | 1.55 | - |  | - | - | - |
| TGF-beta signaling pathway | - | 2.63 | - | - |  | - | - | - |
| Hippo signaling pathway | - | 2.53 | - | - |  | - | - | 2.89 |
| Signaling pathways regulating pluripotency of stem cells | - | 2.31 | - | - |  | - | - | - |
| Renal cell carcinoma | - | 1.99 | - | - |  | - | - | - |
| mTOR signaling pathway | - | 1.99 | - | - |  | - | - | - |
| Thyroid hormone signaling pathway | - | 1.84 | - | - |  | - | - | - |
| Focal adhesion | - | - | - | 7.65 |  | 2.97 | 2.33 | - |
| ECM-receptor interaction | - | - | - | 7.14 |  | - | - | - |
| Proteoglycans in cancer | - | - | - | 5.96 |  | - | - | - |
| Glycolysis / Gluconeogenesis | - | - | - | 5.66 |  | - | - | - |
| PI3K-Akt signaling pathway | - | - | - | 5.57 |  | - | - | - |
| Glycosaminoglycan biosynthesis - heparan sulfate / heparin | - | - | - | 4.63 |  | - | - | - |
| Axon guidance | - | - | - | 4.43 |  | - | 3.41 | - |
| Fc gamma R-mediated phagocytosis | - | - | - | - |  | 4.22 | 3.79 | - |
| Choline metabolism in cancer | - | - | - | - |  | 4.09 | - | - |
| Ras signaling pathway | - | - | - | - |  | 3.81 | - | 3.55 |
| Calcium signaling pathway | - | - | - | - |  | 3.16 | - | - |
| Aldosterone synthesis and secretion | - | - | - | - |  | 3.09 | - | - |
| GnRH signaling pathway | - | - | - | - |  | 2.95 | - | - |
| Regulation of actin cytoskeleton | - | - | - | - |  | 2.88 | - | - |
| Insulin resistance | - | - | - | - |  | - | 3.48 | - |
| Toxoplasmosis | - | - | - | - |  | - | 3.31 | - |
| Morphine addiction | - | - | - | - |  | - | 2.81 | 3.54 |
| Oxytocin signaling pathway | - | - | - | - |  | - | 2.78 | - |
| Purine metabolism | - | - | - | - |  | - | 2.59 | - |
| Glycosaminoglycan biosynthesis - keratan sulfate | - | - | - | - |  | - | 2.46 | - |
| cAMP signaling pathway | - | - | - | - |  | - | 2.36 | - |
| Circadian entrainment | - | - | - | - |  | - | - | 3.49 |
| Non-small cell lung cancer | - | - | - | - |  | - | - | 2.55 |
| MAPK signaling pathway | - | - | - | - |  | - | - | 2.28 |
| GABAergic synapse | - | - | - | - |  | - | - | 2.17 |
| Melanogenesis | - | - | - | - |  | - | - | 2.07 |
| Retrograde endocannabinoid signaling | - | - | - | - |  | - | - | 2.06 |

^a^ The significance of the corresponding pathway enrichment. For each infection group, only KEGG terms within top ten enrichment scores are presented.

^b^ HeV-infected corresponding PaKi or HeLa cell at 6 hpi. For HeV-infected PaKi cells at 6 hpi, only 9 KEGG terms were enriched with p-values < 0.05.

^c^ CedV-infected corresponding PaKi or HeLa cell at 6 hpi.

^d^ HeV-infected corresponding PaKi or HeLa cell at 24 hpi.

^e^ CedV-infected corresponding PaKi or HeLa cell at 24 hpi.

^f^ KEGG term was not enriched in corresponding cells.
